# Influence of child and adult faces with face masks on emotion perception and facial mimicry

**DOI:** 10.1038/s41598-023-40007-w

**Published:** 2023-09-08

**Authors:** Till Kastendieck, Nele Dippel, Julia Asbrand, Ursula Hess

**Affiliations:** 1 Department of Psychology, Social and Organizational Psychology, Humboldt-Universität zu Berlin, Berlin, Germany; 2 Clinic for Psychiatry, Psychosomatics, and Psychotherapy of Childhood and Adolescence, Charité Universitätsmedizin Berlin, Berlin, Germany; 3 Department of Psychology, Clinical Psychology of Childhood and Adolescence, Friedrich-Schiller-University Jena, Jena, Germany

**Keywords:** Psychology, Human behaviour

## Abstract

Emotional mimicry, the imitation of others’ emotion expressions, is related to increased interpersonal closeness and better interaction quality. Yet, little research has focused on the effect of face masks on emotional mimicry and none on (masked) child faces. To address this gap, we conducted an online experiment (*N* = 235, German sample, adult perceivers). Masks reduced emotion recognition accuracy for all expressions, except in the case of anger in masked child faces, where perceived anger was even increased. Perceived interpersonal closeness was reduced for masked happy and sad faces. For both child and adult expressers, masks reduced facial mimicry of happy expressions, with no mask effects for sadness and anger expression. A stronger mask effect on facial happiness mimicry of child faces was mediated by the degree of emotion recognition accuracy. Smiles shown by masked children were not recognized well, likely due to the absence of wrinkles around the eyes in child faces. Independent of masks, sadness shown by children was mimicked even more strongly than when shown by adults. These results provide evidence for facial mimicry of child expressions by adult perceivers and show that the effects of face masks on emotion communication may vary when children wear them.

## Introduction

During the COVID-19 pandemic, face masks became ubiquitous—as did studies on their social effects. Overall, findings suggest that face masks have implications for the success of everyday non-verbal^1^ and verbal^2^ interactions. In emotion research, the vast majority of mask research has looked at effects of face masks on emotion perception. To our knowledge, only one study^3^ focused on emotional mimicry, the spontaneous and largely unconscious imitation of the emotional display of our interaction partner^4,5^. Notably, despite the importance of facial mimicry for social interactions^4,5^, no research to date has looked at mimicry of child faces. In the present study, we assessed the effects of face masks on emotional mimicry, emotion perception, and perceived interpersonal closeness for both adults and children who expressed happiness, sadness, or anger. Hence, this research contributes to our understanding of a central emotion communication process in both the adult-adult and child-adult domain.

### Face masks and emotional mimicry

Face masks as those common during the COVID-19 pandemic occlude the lower part of the face. The lower part of the face is especially diagnostic for smiles, even though more intense smiles are also marked by activity of the orbicularis oculi region, which produces wrinkles around the eye in adults (but due to a still present layer of fat not, or less, in children). By contrast, for other emotions, such as anger, the upper face region is more diagnostic^6^. Thus, while occlusion of the lower face reduces overall emotion recognition accuracy (e.g.,^7,8^), this is not the case to the same degree for all emotions. There is even some evidence that anger is even better recognized when a mask is worn^9^.

However, face masks may affect emotion communication, and especially mimicry, not only due to the partial occlusion of the face but also through their social signal value. For example, there is evidence that face masks can be perceived as a sign of virtue, “a symbol of social solidarity” ^10^, p. e40) but also, conversely, as a threat because they signal contagion or illness^11^. Importantly, there is no conclusive evidence on the valence of the social signal value inherent in masks. We therefore also included measures of attitudes toward mask-wearing as possible moderators.

The social signal value of emotions is central to the emotional mimicry in social context theory^4,5^. Specifically, emotion expressions are perceptually integrated in relation to contextual factors, which then influence reactions such as emotional mimicry. Mimicry depends on and contributes to affiliation, the fundamental motive to connect with others^12^.

Hess and Fischer^4,5^ posit that participants mimic their understanding of other’s facial expressions and not specific muscle movements. Face masks attenuate emotion recognition accuracy but typically people can still detect the emotion expressions well above chance. As such, observers can still deduce the meaning of most emotion expressions, and, consequently, show emotional mimicry. In this vein, Kastendieck et al.^3^ found that masks reduced mimicry for happy but not for sad expressions. The authors suggest that this is because sad expressions signal a strong appeal for succor and empathy^13^.

During the pandemic, the effects of mask-wearing by children were frequently discussed (e.g.,^14,15^). However, little is known about the mimicry of child faces by adults. Even though there are some few studies on children as mimickers (e.g.,^16^), there are none with children as expressers (mimickees). This is a problem as emotional mimicry is typically a mutual process, interaction partners in real-life interactions are typically at the same time expresser and perceiver. Emotional mimicry is a fundamental social regulator in humans and, as such, also fundamentally important for children. Hence, the study of mimicry of child expressers is highly relevant.

Notably, child faces elicit positive affect and sympathy^17^. Hence, it is possible that these juvenile faces signal a stronger appeal for empathy thereby enhancing mimicry because emotional mimicry is an important correlate of affective empathy^18^. By contrast, it should be noted that, in many contexts, children are less expected to wear masks. As such, masked children as compared to masked adults are a less common stimulus that, for this reason, may create a feeling of strangeness, which in turn would reduce mimicry. Yet, even though face masks may be less common for children, they were and to some degree still are a fact of life. Children wear masks in a variety of social contexts (e.g., hospitals, doctor’s offices, etc.) and did so especially due to mask mandates during the COVID-19 pandemic. The study took place from August 2021 to March 2022 and during this time, the mask regulations for children of our stimulus age range (8–12) were in effect in Germany where the study took place as well as in many other countries (as suggested by the WHO, https://www.who.int/news-room/questions-and-answers/item/q-a-children-and-masks-related-to-covid-19). Overall, it is therefore of importance to better understand how adult perceivers affectively empathize with child expressers during pandemic times and beyond.

### Face masks and emotion perception

Given the relatively short time since the start of the COVID-19 pandemic (i.e., relative to the history of emotion research), there is already an abundance of studies on face masks and emotion perception (e.g.,^3,7,19–24^). The overall consensus is that face masks can reduce perceived intensity and to a lesser extent recognition rates of basic emotions. Specifically, emotion expressions with lower facial diagnostic regions, such as happiness and sadness, appear to be impaired more than emotion expressions with upper facial diagnostic regions, such as anger^20^. Other evidence suggests that there may be no mask effects on anger perception at all (^25,26^; see^27^, for an exception) or that there is even more anger (and fear) accuracy when a mask is present^9^. Hence, effects of anger perception appear to be more ambiguous, which could be expected for anger mimicry too.

However, it is possible that, with ongoing mask exposure, people may learn to better cope with masks. This notion is supported by eye-tracking data that suggests shifts in attention to different facial areas when the mask is present^28,29^. In line with the fact that people are relatively good at judging mental states from the eye region alone (^30^; but see^31^, for evidence of mask effects on theory of mind), this is especially relevant for the mimicry of happiness. As noted above, more intense smiles are characterized by wrinkles in the eye region—due to activity of the orbicularis oculi pars lateralis—which remain visible even when a mask is worn. Also, when stimuli are dynamic, observers can see the cheeks move up in smiling.

In sum, we predict a reduction of emotion perception and emotional mimicry based on previous research on mask effects. While it is also possible that mask-related learning processes may change emotion processing in the course of the pandemic, to date, there is no longitudinal evidence either way. Further, whereas intense smiles shown by adults are usually recognizable through the “wrinkles around the eyes” due to activity of the orbicularis oculi muscle in addition to the pushing up of the cheeks, children’s faces do not wrinkle as much, if at all. Hence, children’s smiles should be more difficult to decode when a mask is worn and hence mimicked to a lesser degree.

### Face masks and interpersonal closeness

Face masks have been found to reduce perceived interpersonal closeness^3,8,32^, unless contagion danger is salient^8^. However, to the degree that face masks are perceived positively, the negative effect of face masks on interpersonal closeness could be lessened. We therefore assessed attitudes toward mask-wearing as potential moderators for the effect.

### Goal of the present study

In sum, the goal of the present study was to investigate whether (a) masks have an impact on emotion perception for both child and adult target faces and (b) masks reduce affiliative mimicry of happy and sad expressions as well as (c) perceived interpersonal closeness; (d) we further explored whether these effects extend to anger stimuli and whether target age (child vs. adult) moderates these relationships. (e) As a positive or negative attitude toward masks can affect affiliation toward mask wearers and thereby emotion perception and mimicry, we included three measures of attitudes toward mask-wearing as potential moderators.

Because anxiety and depression can affect emotion processing (e.g.,^33^), we included self-report measures of anxiety (General Anxiety Disorder-7 Scale, GAD-7;^34^) and depression (Brief Patient Health Questionnaire, PHQ-9;^35^) as possible moderators. The research questions and approaches toward the analysis were recorded in a preregistration which can be found at https://osf.io/cqre5.

#### Hypotheses

Specifically, we hypothesized that face masks, as compared to an unmasked condition, will lead to reductions in perceived emotion intensity and recognition performance, emotional mimicry of happy but not sad expressions, and perceived interpersonal closeness. We had an explorative stance toward anger and target age moderation effects with no directed hypotheses.

## Results

### Data analysis strategy

For the analyses of the full experimental design, we used the *lmerTest*^36^ extension for *lme4*^37^. Participant id was used as a cluster variable with random intercept; random slopes were not specified due to non-convergence. For global evaluation of interaction effects in the full design models, we used type III sum of squares F tests. To test specific hypotheses, we conducted contrast analysis: For the rating data, we tested simple effects and for the facial activity data, we tested custom (or focused) contrasts, both via *emmeans*^38^. Bonferroni correction was used to account for multiple testing (results were not substantially different when more sophisticated methods, such as multivariate t,^39^, were used). We used two-tailed testing.

As for the moderation effects, due to the explorative nature of the analyses, we wanted to be more conservative and changed the criterion for statistical significance for moderation analyses with a continuous moderator from 0.05 to 0.01. We further explored the main effects of participant gender, target sex, and participant age (for interactions, we found some minor effects in focused analyses but overall inconsistent results under conditions of lack of power). We concluded from these control variable analyses that our main results of our core analyses were robust.

The core analyses in this study revolve around a mask*targetage interaction, which, overall, remained intact when moderators were included (see Supplementary Information document linked below in this article and uploaded to the Supplementary Information folder at https://osf.io/smqpk/?view_only=f2681fb953f54a149238efaf243bb9c0). Thus, we report these analyses as they were pre-registered, but only in the Supplementary Information. We departed from the preregistration for the mimicry analysis since we could not include type of emotion as a factor as the model did not converge. We therefore conducted separate analyses for each emotion. This decision was supported by the notion that mimicry of the different emotion expressions may follow different mechanisms^40,41^. We report the main analyses below, for detailed results and code, see R Markdown (see HTML document uploaded to the analysis folder, and please navigate through floating table of content at top left, at https://osf.io/smqpk/?view_only=f2681fb953f54a149238efaf243bb9c0).

### Emotion perception

The following results for emotion perception are divided into hit rate (emotion recognition performance as the proportion of correctly identified expressions) and the emotion intensity rating for the target scale (e.g., the happiness rating for happy expressions).

### Hit rate

**Figure 1:**
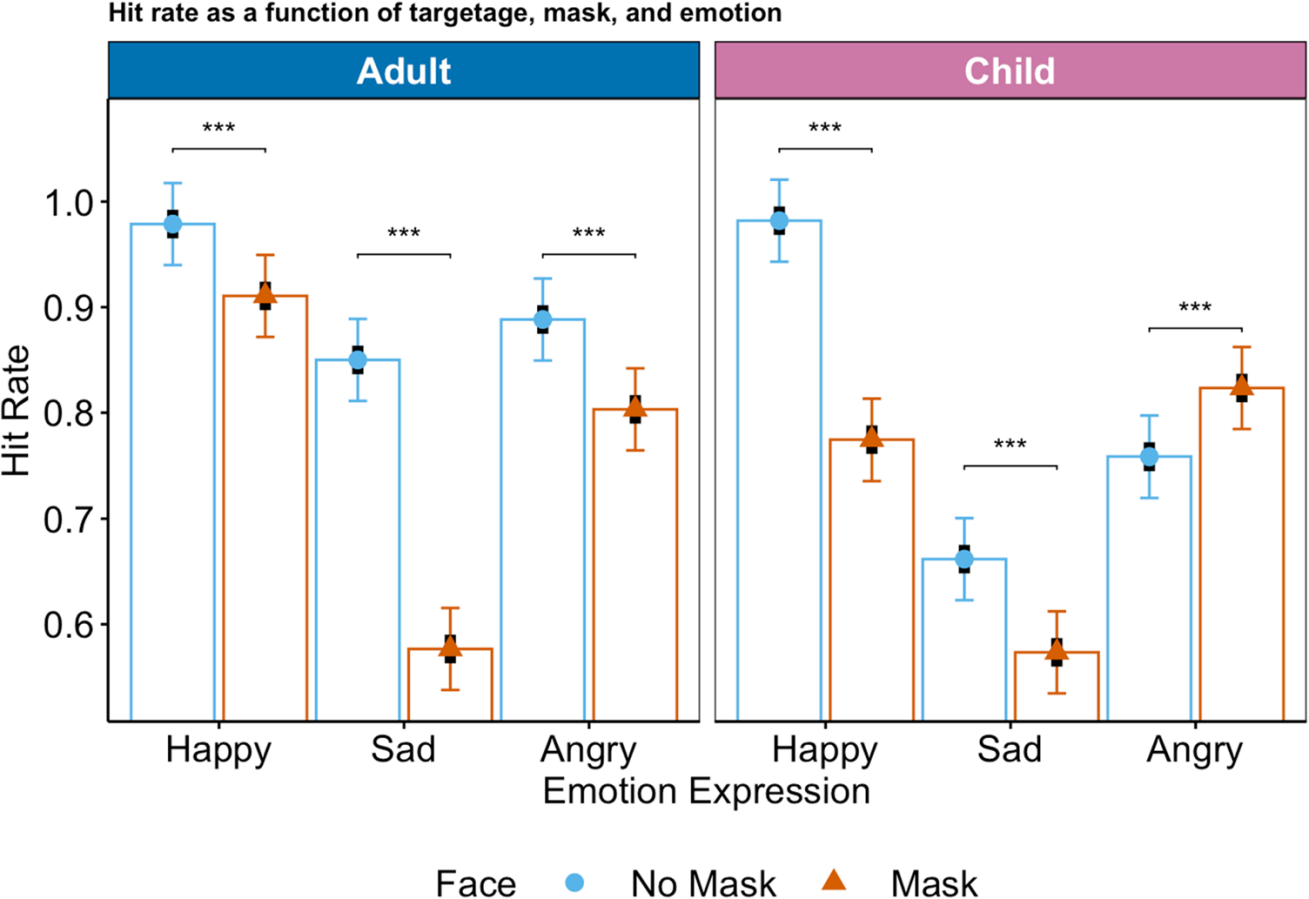
Hit rate as a function of mask, emotion expression, and target age. Symbols (circle: no mask, triangle: mask) represent estimated marginal means, black bars represent standard errors, colored bars represent 95%-confidence intervals. The possible values range from 1 to 100%.

A model with the fixed factors mask (reference: no mask), emotion (reference: happy), and target age (reference: adults) with hits as the dependent variable revealed that all lower-order effects were qualified by a significant three-way interaction, *F*(1, 11034) = 55.64, *p* < 0.0001. For the estimated marginal means plot, please see Fig. 1 (for the observed means plot, please see R Markdown).

Contrast analyses (simple effects) indicated that the recognition rate of sadness was reduced when masks were worn and the difference between masked and unmasked faces was larger for adult targets (85% vs. 58%, *p* < 0.0001, Cohen’s *d* = 0.75) than child targets (67% vs. 57%, *p* < 0.0001, Cohen’s *d* = 0.24). The hit rate for happiness was only slightly reduced for adult expressers (98% vs. 91%, *p* < 0.0003, Cohen’s *d* = 0.19), whereas there was a substantial reduction in hit rate for masked child targets (98% vs. 77%, *p* < 0.0001, Cohen’s *d* = 0.57). For anger, a higher hit rate for masked versus unmasked child targets (82% vs. 76%, *p* < 0.0007, Cohen’s *d* = − 0.18) emerged, with the reverse pattern for adult targets (88% vs. 80%, *p* < 0.0001, Cohen’s *d* = 0.23).

### Intensity

**Figure 2:**
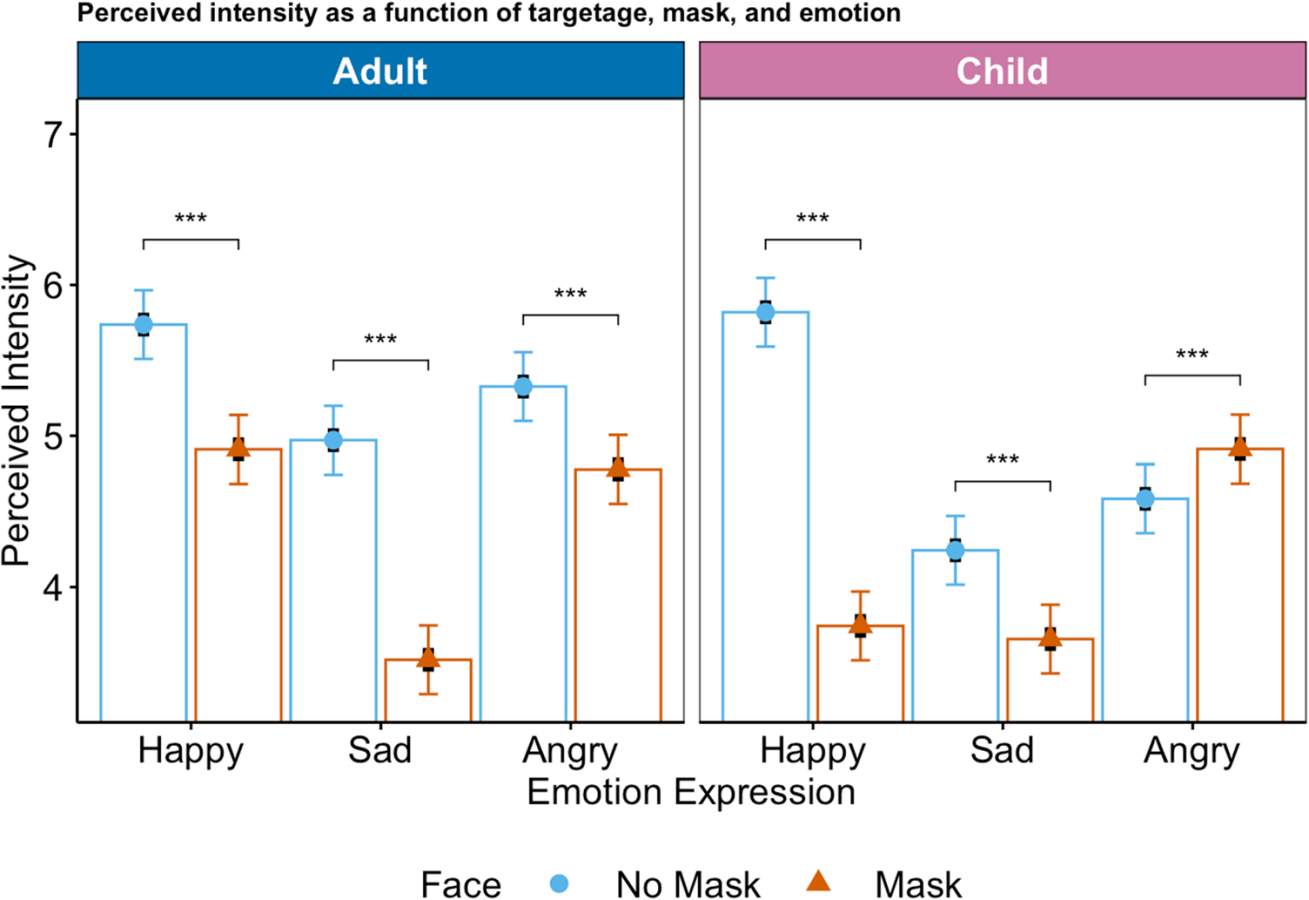
Rated emotion intensity as a function of mask, emotion expression, and target age. Symbols (circle: no mask, triangle: mask) represent estimated marginal means, black bars represent standard errors, colored bars represent 95%-confidence intervals. The response scale ranged from 1-7.

For the emotion intensity ratings as well, all lower-order effects were qualified by a significant three-way interaction, *F* (1, 11034) = 136.18, *p* < 0.0001. For the estimated marginal means plot, please see Fig. 2 (for the observed means plot, please see R Markdown).

Contrast analyses indicated that when expressers wore a mask, the target emotion intensity was rated significantly lower for both happy (adult: *p* < 0.0001, Cohen’s *d* = 0.51, child: *p* < 0.0001, Cohen’s *d* = 1.29) and sad faces (adult: *p* < 0.0001, Cohen’s *d* = 0.09, child: *p* < 0.0001, Cohen’s *d* = 0.37). For angry faces, the perceived emotion intensity was lower when adults wore a mask (vs. no mask; *p* < 0.0001, Cohen’s *d* = 0.34) but higher when children wore a mask (vs no mask; *p* < 0.0001, Cohen’s *d* = − 0.20).

### Perceived interpersonal closeness

**Figure 3:**
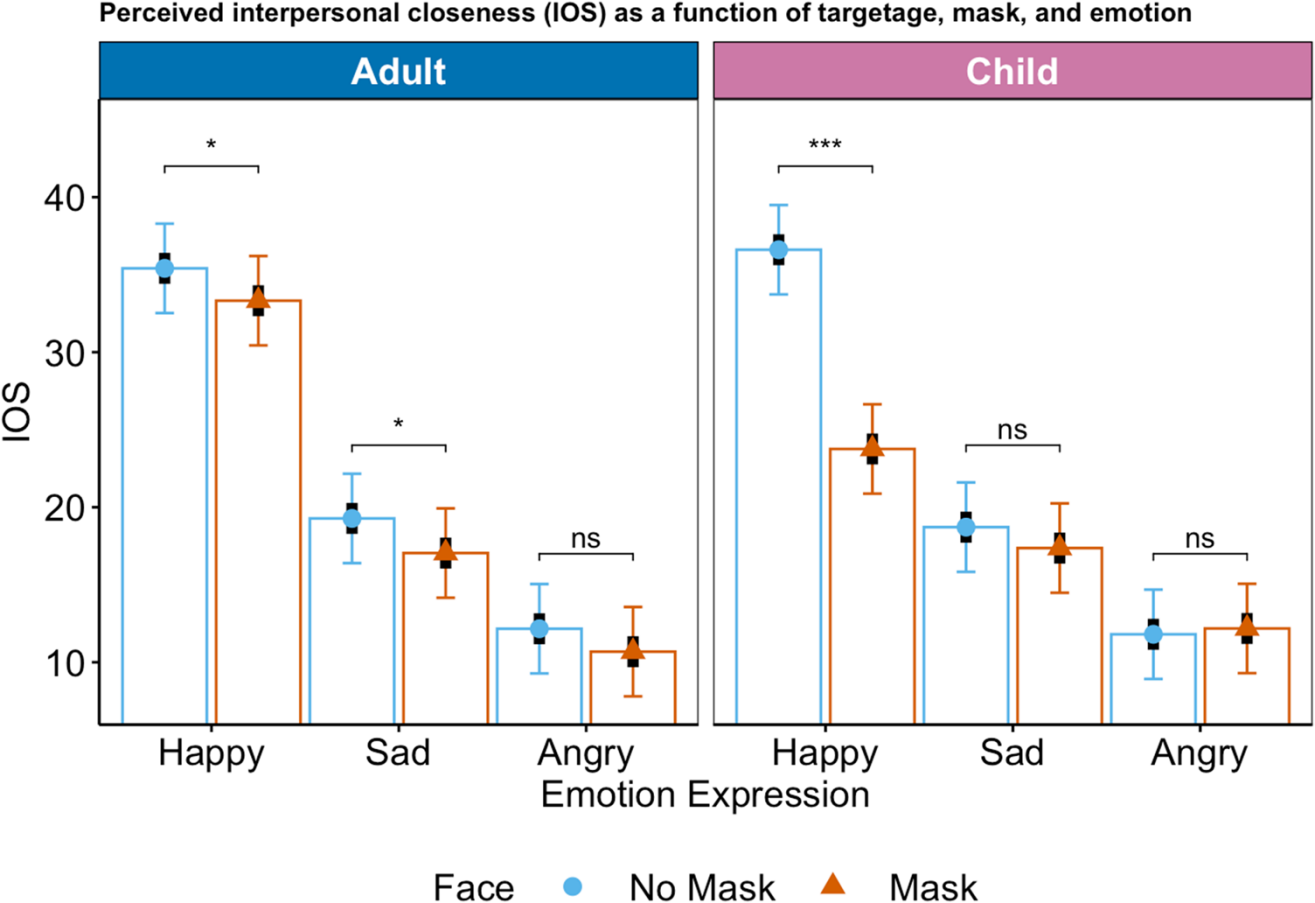
Perceived interpersonal closeness (IOS) as a function of mask, emotion expression, and target age. Symbols (circle: no mask, triangle: mask) represent estimated marginal means, black bars represent standard errors, colored bars represent 95%-confidence intervals. The response scale ranged from 1-100. The scale was adapted as a slider from no overlap to more overlap.

For interpersonal closeness, all lower-order effects were qualified by a significant three-way interaction, *F*(1, 11034) = 45.4, *p* < 0.0001. For the estimated marginal means plot, please see Fig. 3 (for the observed means plot, please see R Markdown).

Contrast analyses indicated that when child expressers of happiness wore a mask, interpersonal closeness was particularly low (vs no mask; *p* < 0.0001, Cohen’s *d* = 0.81). A similar, smaller, but still significant effect emerged for adult happy (*p* = 0.028, Cohen’s *d* = 0.13) and sad (*p* = 0.015, Cohen’s *d* = 0.14) targets. No significant differences between mask vs no mask emerged for anger expressions.

### Facial mimicry

Facial mimicry was indexed by either (a) a pattern score [the difference between the Mean(AU12 Lip corner puller + AU06 Cheek raiser) and AU04 Brow furrow), for more details, see “Methods” section] significantly larger than zero or (b) scores for onset and apex, respectively, that are significantly larger than scores for responses to neutral expressions. Happiness mimicry was indexed by a positive pattern score and sadness and anger mimicry by a negative pattern score. Values significantly above zero indicate a matching expression by participants, values around zero indicate no expression, and values below zero indicate a counter-expression.

The main model included the fixed factors segment (reference: neutral expression/still face), mask (reference: no mask), and target age (reference: adult) to predict the positive expressivity score for happy expressers and the negative expressivity score for sad expressers and angry expressers, respectively.

### Happy expressions

**Figure 4:**
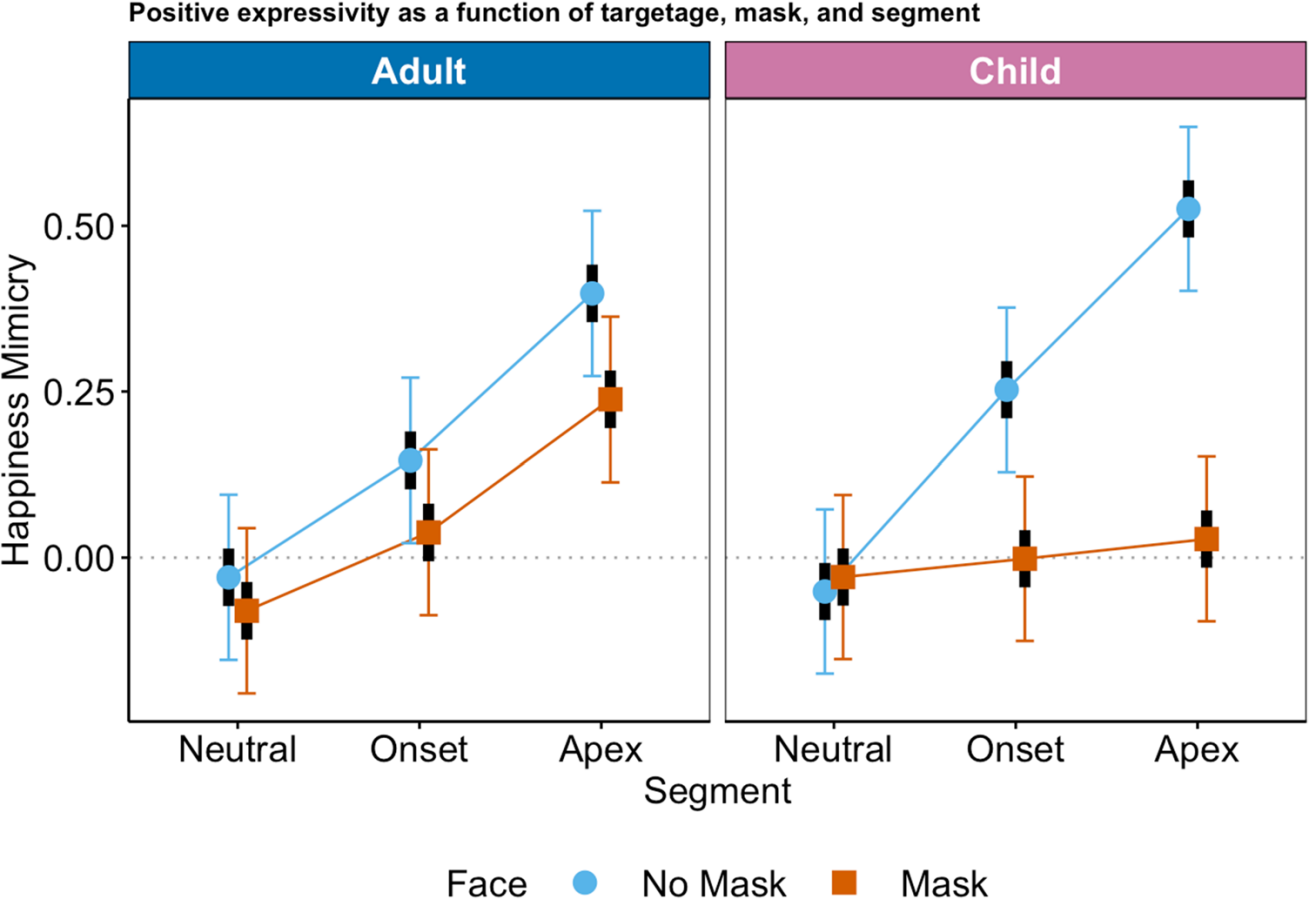
Facial activity in response to happy expressions (happiness mimicry) as a function of mask, segment, and target age. Symbols (circle: no mask, triangle: mask) represent estimated marginal means, black bars represent standard errors, colored bars represent 95%-confidence intervals.

All lower-order effects were qualified by a significant mask*segment*targetage interaction, *F(*1, 9297) = 6.82, *p* = 0.001. For the estimated marginal means plot, please see Fig. 4 (for the observed means plot, please see R Markdown). Comparisons of estimated marginal means to zero indicated that for unmasked expressers, mimicry was present at both the onset (adult: 95% CI [0.02, 0.27], child: 95% CI [0.13, 0.38]) and the apex of the expression (adult: 95% CI [0.3, 0.52], child: 95% CI [0.40, 0.65]), whereas for masked expressers, mimicry was significantly different from zero only at apex and only for adult expressers, 95% CI [0.11, 0.36]. The facial reaction at the neutral segment was not different from zero, indicating that there were no facial reactions to neutral faces.

For adult expressers, custom contrasts revealed no significant differences between unmasked and masked expressers, neither at onset nor at apex (at apex, the difference was just not significant, estimate = 0.16, adjusted *p* = 0.059, unadjusted *p* = 0.03, Cohen’s *d* = 0.14). For child expressers, the response to unmasked expressers was significantly higher than toward masked expressers for onset (child, onset: estimate = 0.25, *p* < 0.001, Cohen’s *d* = 0.23) and apex (child, apex: estimate = 0.50, *p* < 0.001, Cohen’s *d* = 0.45).

Regarding the comparison of onset and apex vs the neutral segment, facial positivity was higher at onset and apex than at neutral for unmasked expressions (adult, onset: estimate = 0.18, *p* = 0.022, Cohen’s *d* = 0.16; child, onset: estimate = 0.30, *p* < 0.0001, Cohen’s *d* = 0.28; adult, apex: estimate = 0.43, *p* < 0.0001, Cohen’s *d* = 0.39; child, apex: estimates = 0.58, *p* < 0.0001, Cohen’s *d* = 0.52) but for masked expressions this was the case only for adult expressers at apex (adult, apex: estimate: 0.30, *p* < 0.0001, Cohen’s *d* = 0.27).

In sum, mimicry of happy expressions emerged consistently for both adult and child unmasked expressers. Whereas adult masked expressers were still mimicked at the peak of expression, mimicry was entirely absent for masked child expressers.

### Sad and anger expressions

For both sad and angry expressions, no interaction effects emerged, that is, mimicry was not differentially affected by masks for either adult or child expressers. For sadness, a significant effect of targetage emerged, *F*(1, 9340) = 11.23, *p* = 0.001, such that sadness in child faces was mimicked more strongly than in adult faces.

For both sadness and anger and in both target age groups, a pattern congruent with mimicry emerged (sadness: segment main effect, *F*(1, 9335) = 9.55, *p* < 0.0001); anger: segment main effect, *F*(1, 9255) = 14.80, *p* < 0.0001; see R Markdown for statistical analyses and plots).

### The mediating role of emotion perception in the mask-mimicry relationship

**Figure 5:**
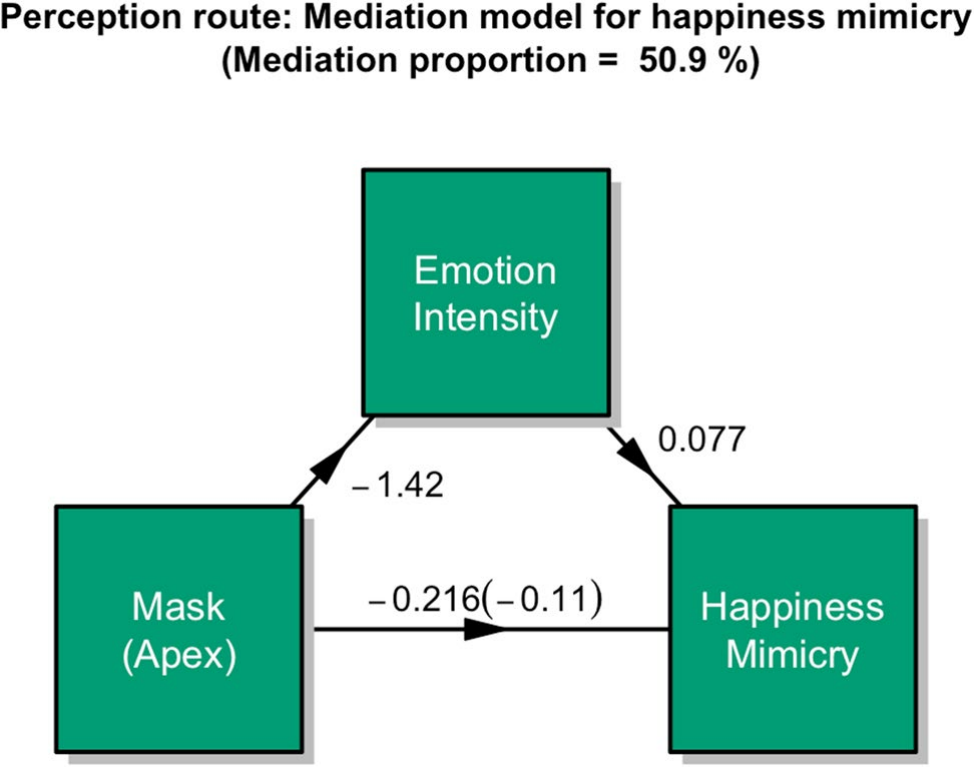
Multilevel mediation model for happiness mimicry.

Kastendieck et al.^3^ reported that differences in the mimicry of masked vs unmasked happy expressions were mediated by differences in the perceived intensity of happiness. These findings could be replicated for both adult and child faces, as shown in Fig. 5 (overall: mediation proportion 55.6%). A separate analysis showed that the effect was twice as strong for child (63%) than for adult (28.4%) faces. This result supports the notion that facial mimicry of happiness is dependent on the appraisal of the face as showing happiness as suggested by the Emotional Mimicry in Context Theory^4,5^. Moreover, it supports the view that adult perceivers had more trouble recognizing happiness in masked child faces, possibly due to the absence of eye wrinkles. Since masks did not reduce the mimicry of sad and angry faces, no mediation models were assessed.

### Exploratory analysis: emotion perception

The main analyses of the emotion ratings revealed reductions in perceived intensity for masked faces (except for anger in masked child faces). This raises the question of whether this implies that overall intensity ratings are lower as found by Hareli et al.^42^ or whether participants rated other emotions instead. Kafetsios and Hess^43^ distinguish in this context between accuracy (the rating of the correct emotion) and bias (the ratings on all other rated emotions). The intensity ratings reported above correspond to accuracy. Ancillary analyses were conducted to assess bias.

Higher overall bias (i.e., average across all non-target scales) for masked (vs unmasked) faces was found for child happy expressions and for adult sad expressions. Similar but smaller mask-no mask differences were significant also for the remaining factor level combinations except anger in children. These findings point to reduced signal clarity of masked faces, which may lead to errors in ratings.

Specifically, participants rated masked faces more negatively (i.e., mean of negatively valenced emotions sadness, anger, fear, and disgust), again except in the case of anger in children. Notable were, in particular, higher ratings of anger for happy masked child faces.

## Discussion

The goal of the present research was to assess the effects of face masks on emotion communication in terms of both emotion recognition and facial mimicry for both adult and child faces expressing happiness, sadness, or anger.

The results show that both the perception and mimicry of happiness is impacted by the presence of masks which is even more the case for children. The effect on mimicry was mediated by the reduction in the perceived intensity of the expression.

These findings replicate results by Kastendieck et al.^3^ and extend the findings to children. One explanation why happiness communication by children was impacted more strongly by masks lies in the morphology of children’s faces. Due to the still existing layer of fat under the skin, children’s faces do not wrinkle when the orbicularis oculi muscle is activated. Yet, the wrinkles around the eyes are a major cue for smiling when wearing masks. Since we used video stimuli, an additional cue—the pushing up of the cheeks in smiling—was still present. However, this cue is less salient, which explains the much stronger reduction in decoding accuracy and perceived intensity for child versus adult happy faces. Moreover, a second reason may be that in many contexts, children are less expected to wear masks and hence adults are less trained in perceiving masked children. In addition, perceived interpersonal closeness was much reduced when children wore masks while showing happiness. This is likely due to a misperception of happiness as anger. In fact, with the absence of wrinkles, the reduced eye size, which is the effect of pushing the cheeks up in a smile, can resemble the squinting that is part of an anger expression. Given the importance for interpersonal closeness for mimicry (e.g.,^40^), this effect might also have contributed to the reduction in mimicry.

In contrast, even though recognition accuracy was affected by the mask, more for adults than for children, sadness was mimicked independent of masks, again replicating findings by Kastendieck et al.^3^. Kastendieck et al.^3^ explain the preserved mimicry for sadness expression by the strong appeal function of this expression to empathize or feel with the other^44^. The target age main effect we found suggests that children who express sadness were mimicked more than adults, which may be explained by the juvenescence effect of child faces^15^ which amplifies the appeal for succor inherent in sad faces^44^. Anger expressions were also mimicked independent of the condition. Similar to what Grenville and Dwyer^9^ found for adult expressers, we found for child expressers that anger expressions were perceived as more intense when the mask was present. Thus, children were not generally mimicked less than adults but only when they showed happiness and wore a mask, whereas they may be even mimicked more when they express sadness.

Overall, we did not find strong effects of attitudes toward mask-wearing on either emotion perception or mimicry (see Supplementary Information). Yet, in the masked happy conditions, higher mask compliance was associated with less emotion rating bias. This suggests that only the behavioral endorsement of mask wearing, but not more abstract endorsements, had an—albeit rather limited–effect on the perception of masked others.

In sum, the present study replicated previous findings regarding the effect of mask-wearing on facial mimicry and emotion perception for adults and extended the findings to children. These findings are especially important because there is a stark lack of research on mimicry of child expressers. The findings suggest that the mimicry of happiness, which plays a very important role for smooth and affiliative interactions^41^ and is a correlate of affective empathy^18^, is negatively affected by face masks and this more so for child than adult expressers. This is also reflected by the strong reductions in perceived interpersonal closeness toward masked children expressing happiness. On the other hand, sadness (also an indicator of affiliative empathy) was mimicked even more in response to child faces, independent of face masks.

These findings have implications with regard to successful emotion communication when advocating mask-wearing especially for children. However, one limitation of the present study is that only facial expressions were considered. In real life interactions, happiness would also be expressed vocally and through posture. As the mediation analyses suggest that the reduction in happiness mimicry is due to the reduced perceived intensity of happiness, it is likely that, when happiness is recognized through other channels, mimicry would be reinstated through cross-modal mimicry^45^. Further, it is possible that, over time, perceivers learn to better compensate for the effects of masks, for example, due to shifts in attention to the eye region.

The present study included children as expressers, but children as perceivers also should not be neglected. Specifically, there is evidence that children have particular problems with holistic face processing^46^ and emotion reading when a mask is present (e.g.,^13,47–51^), whereas adolescents on the other hand may be particularly good at recognizing sadness and anger^52^. However, how masks may affect mimicry by children has not been assessed.

Furthermore, future studies could shed more light on the positive side of the social effects of masks and how these relate to the disadvantages. For example, attractiveness ratings may even increase when the mask is present^21^ and higher levels of attractiveness are associated with more perceived interpersonal closeness (^53^, manuscript submitted for publication).

## Conclusion

In sum, the present study suggests that face masks do not only impede emotion perception and interpersonal closeness but also facial mimicry of happiness, especially when the wearer is a child. These findings highlight that children are an especially vulnerable group when considering how they may be perceived by adults during the pandemic. Without the capacity to recognize and mimic emotions, and feel close to another person, the interactional flow is decreased, which, especially for children, has serious potential social costs during pandemic times and beyond. However, it should be noted that this restriction does not mean that masks fully impede empathy (as indexed by facial mimicry). This is because sadness and anger expressions were mimicked independent of masks. Sadness in children was even mimicked more strongly. Together with the fact that multiple emotion communication channels are active simultaneously, interactional flow may thus be maintained nonetheless.

## Methods

### Power considerations

For the focal emotional mimicry analysis, a power simulation using the R package *simr*^54^ with a 2*2*3 design (mask * target age * segment) suggested that N > 200 allows for 80% power (see power analysis and power curve plot see power analysis and power curve plot uploaded to the analysis folder at https://osf.io/smqpk/?view_only=f2681fb953f54a149238efaf243bb9c0). Since we conducted an online study, participants were filmed using their webcams and face videos were automatically analyzed. Thus, we anticipated a relatively high loss of data. We therefore decided to oversample to compensate for likely technical problems (e.g., faulty or slow internet connections, problematic browser settings, or poor camera resolution).

### Participants

The final rating data sample consisted of data from 235 participants (97 women) with a mean age of 29.6 (*SD* = 10.8; age range 18–70), who reported having seen the video stimuli (one was excluded as they did not) and for whom at least one video upload was successful (21 were excluded because none were uploaded). Of the 11280 video stimuli across participants, only 186 video uploads were not successful (0.02%). The facial activity sample consisted of 206 participants (for 8 participants, no facial activity data was available), just over our minimum target of 200. The mean task time was 48 minutes (advertised as a 40-minute task). Prolific participants received €9.47 on average for their participation, which is classified as *good* payment in Prolific. Participants’ anonymity was protected by the use of a Prolific id. Non-Prolific participants, who were recruited via Instagram posts and subsequent separate email contact as well as university networks, received an expense allowance only (i.e., 5€; somewhat larger discrepancy due to a price raise in Prolific during the course of the study). Their anonymity was protected by a participant code. Informed consent was obtained from all participants prior to their participation. The study (including the experimental protocol and methods) was approved by the Institutional Review Board (Ethics committee, proposal number #2021-10R1) of the Institute of Psychology at the Humboldt University Berlin and conducted in accordance with the Declaration of Helsinki and the Guidelines for Safeguarding Good Research Practice of the German Research Foundation. All participants participated via the platform Sosci Survey during August 2021 to March 2022.

### Materials

**Figure 6:**
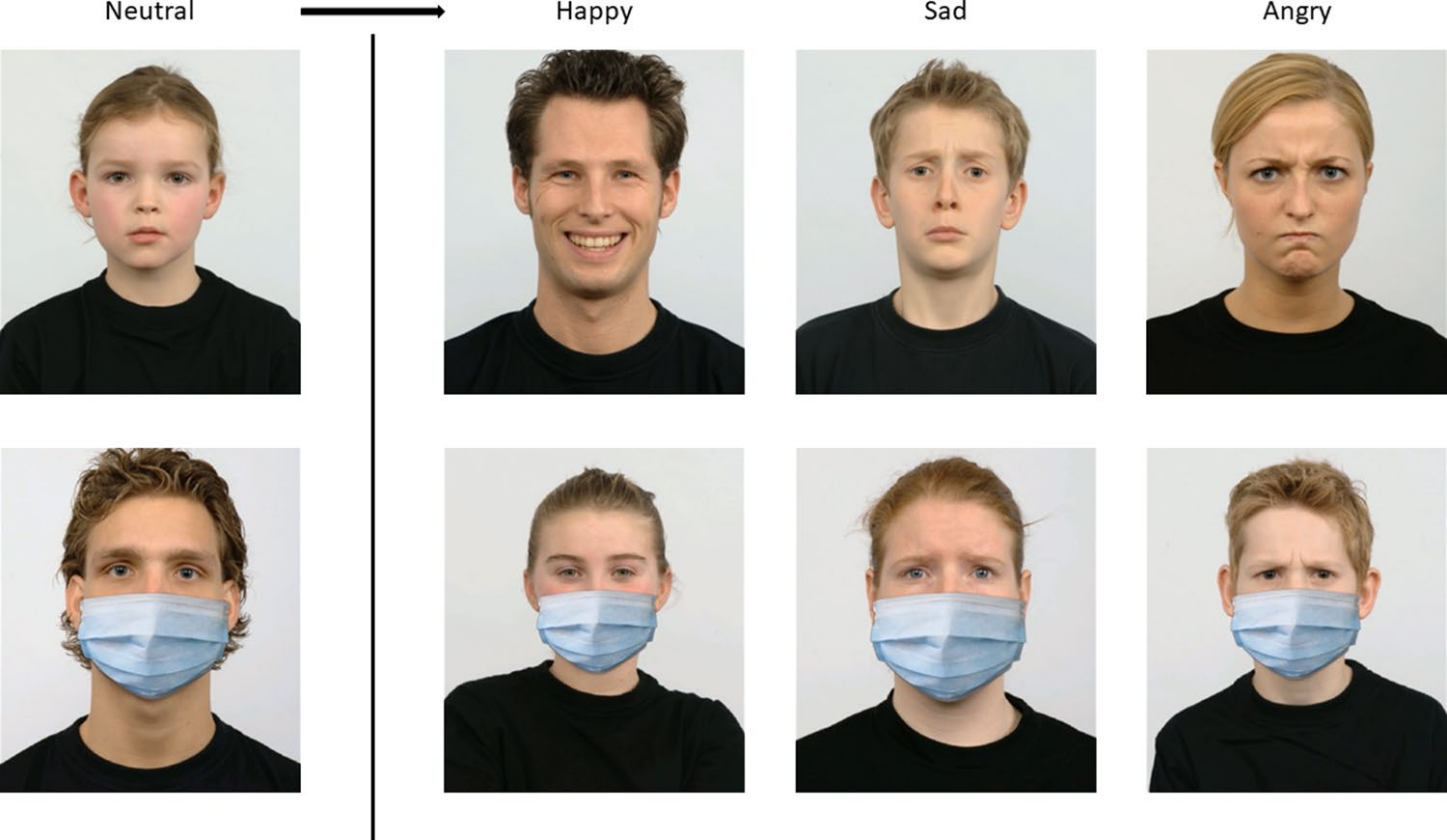
Examples of happy, sad, and angry expressers with and without masks. Facial video stimuli were adapted, with permission, from the Radboud Faces Database^55^ and morphed to achieve dynamic stimuli. Depicted are frames at *the apex* or peak of expression.

For the face stimuli, four each female adult, male adult, female child, male child actors from the Radboud Faces Database^55^ were selected, each showing a static happy, sad, or angry expression. Since dynamic stimuli have been found to be superior to static images when studying facial mimicry^56^, we used FantaMorph 5, to create dynamic expressions by morphing from neutral to apex expressions. The resulting video stimuli were edited using segmentation within an augmented reality software (Lens Studio by Snap Inc.), such that a surgical face mask was added for the masked condition. The mask was attached to the face using the face tracking function of Lens Studio to model a dynamic interplay of the face and mask, allowing the mask to move naturally with the moving face (see stimuli folder at https://osf.io/smqpk/?view_only=f2681fb953f54a149238efaf243bb9c0). For examples of the stimuli at apex, please see Fig. 6. The actors or, if applicable, their legal guardians, from this pre-validated standard face database provided consent to publish the images.

The videos had a length of 6.5 (happy, angry) or 7.5 (sad) seconds and first showed a fixation cross (1.5s), then the neutral face (2s), the developing of the expression (happy/angry: 1.5s; sad: 2.5s), and finally the apex expression (1.5s). Sadness onset was slightly longer as the natural development of a sad expression configuration tends to take longer. The final stimulus set consisted of 48 videos in total. These were comprised of 2 (target sex) × 4 (actors, i.e., per target sex and target age) × 3 (emotion expression) × 2 (target age) with masked vs. unmasked trials being distributed evenly across the stimuli overall. The presentation had the restriction that the same target sex was not presented more than twice in a row. We made sure that the distribution of the factor level combinations was balanced for the participants. The full randomization plan and the full set of stimuli can be found in the procedure folder at https://osf.io/smqpk/?view_only=f2681fb953f54a149238efaf243bb9c0.

### Procedure and measures

Participants were informed that they will see 48 short videos and that participation in the experiment is only possible if they have a webcam-enabled computer/laptop and agree to a webcam recording of their face during the experiment. Informed consent included standard details on compensation, confidentiality, and contact information as well as detailed information on (video) data storage and processing. Participants who agreed to participate were instructed to set up their webcam to allow recording, to arrange sufficient lighting, and to refrain from eating or covering their face during the experiment. Finally, participants provided socio-demographic information.

Participants then saw the video stimuli while their facial activity was recorded. Following each video, using 7-point Likert scales, participants rated the targets’ emotion expressions using an emotion profile (happiness, sadness, fear, anger, disgust, and surprise), as the basis for the perceived intensity and hit rate measures, and to indicate how (socially) close they felt to the person shown using an adapted version of the Inclusion of Other in the Self-scale (IOS,^57^, see Supplementary Information for details), as a measure of perceived interpersonal closeness. The rating on the target emotion in the emotion profile (i.e., happiness for happy faces, sadness for sad faces, and anger for angry faces) served as the perceived intensity measure. For the hit rate, a hit was counted if the rating on the target emotion was higher than the ratings of all other emotion terms in the emotion profile. Following the video task, participants were asked mask-related questions, i.e., mask compliance, mask voluntariness, and an adaptation of the Coronavirus Social Distancing Scale^58^, adapted to the context of mask-wearing named mask endorsement (see Supplementary Information for details). In addition, participants were asked to estimate the perceived physical distance to the persons they had seen (overall rating on all targets in feet and inch) and to answer questions regarding the extent to which they felt affected by COVID-19, whether they or someone close to them was diagnosed with COVID-19, what type of face mask they wear themselves during the pandemic, and geographical data. These latter data were not used for the present report. Descriptively, the vast majority of participants reported that they wear masks (cloth/homemade = 3, surgical = 62, N95/valve = 162, other, e.g., scarf = 4); only four said that they do not. Participants showed strong mask compliance (single item, 5-point scale on how often participants wear a mask at places designated for mask-wearing, such as public transportation; *M* = 4.85, *SD* = 0.52, skew = − 4.25, kurtosis = 20.6) but only moderate trait mask endorsement [9-item instrument, 5-point scale, based on An et al.’s^58^ scale, *M* = 3.06, *SD* = 1.05, skew = − 1.85, kurtosis = 3.22] and mask voluntariness (single item, 5-point scale, on the degree to which participants wear a mask even if it was not mandatory; *M* = 3.1, *SD* = 1.24, skew = − 0.24, kurtosis = − 0.98). More than half of participants (135/235, 57%) reported they or someone in their immediate social circle had been sick with COVID-19. On average, participants felt moderately affected by the pandemic (*M* = 3.92, *SD* = 1.61, skew = − 0.2, kurtosis = − 1.03, 7-point scale). We also measured participants’ self-reported propensity to interact with the targets. However, the overlap with IOS was quite high, *r* = 0.68, 95% CI [0.67, 0.69] and the results similar, we therefore do not report this variable in the framework of this manuscript (for more details and results see R Markdown).

On each page of the procedure, participants were given the opportunity to opt-out and discard their data. Finally, participants were informed about the purpose of the experiment, thanked for their participation, and received the necessary code for payment.

### Facial behavior analysis

The uploaded videos were analyzed using the open-source facial behavior analysis toolkit OpenFace 2.0^59^. OpenFace analyzes facial activity in terms of facial action units as classified in the Facial Action Coding System^60^. Frames with detection confidence lower than 75% were excluded^61^. The mean confidence across all used frames was 97% (*SD* = 0.03). Based on a visual check, OpenFace data from 22 participants had to be excluded as they showed insufficient adherence to task instructions (e.g., talking or eating during the task), used insufficient lighting, or obstructed their face.

### Data preparation

Based on the OpenFace data, facial mimicry was assessed using facial action units AU4 (eyebrows drawn together), AU6 (wrinkles in the corner of the eyes), and AU12 (lip corners pulled up). Baseline-corrected, within-subject z-transformed scores were calculated to control for participants’ general expressiveness. The fixation cross (1.5 s) before each stimulus served as a dynamic baseline. With these transformed scores, a positive pattern score was computed, as described by Hess et al.^62^ for facial EMG, which indicates the contrast between the average activity of zygomaticus major (or AU12 in OpenFace) and orbicularis oculi pars lateralis (AU6), minus the activity of corrugator supercilii (AU4). The converse contrast formed the negative pattern score. Frames of the uploaded videos were divided into segments corresponding to those of the stimulus material: neutral face, onset (developing expression), and apex (peak of expression). The segment factor represents the dynamic of the stimuli across time while allowing categories that reduce data complexity.

### Supplementary Information

Supplementary Information.

## Data Availability

Datasets, HTML of a detailed R Markdown, HTML of a power simulation, and videos of example stimuli can be found at https://osf.io/smqpk/?view_only=f2681fb953f54a149238efaf243bb9c0. The datasets can also be found at Harvard Dataverse at 10.7910/DVN/T5XCQA.
