# Supplementary material for: Influence of child and adult faces with face masks on emotion perception and facial mimicry
Source: Sci Rep. 2023 Sep 8;13:14848. doi: 10.1038/s41598-023-40007-w (PMC10491609; doi:10.1038/s41598-023-40007-w)
Supplement: Supplementary file 1 — Supplementary Information. [file 41598_2023_40007_MOESM1_ESM.pdf]

Supplementary Information: Moderation Analyses

Influence of Child and Adult Faces With Face Masks on Emotion Perception and Facial Mimicry

Till Kastendieck*[1]
Nele Dippel[2]
Julia Asbrand[2,3]
Ursula Hess[1]

[1]Humboldt-Universität zu Berlin, Institute of Psychology, Department of Social and Organizational Psychology
[2]Humboldt-Universität zu Berlin, Institute of Psychology, Department of Psychology and Psychotherapy of Children and Adolescents
[3]Friedrich-Schiller-University Jena, Department of Psychology, Clinical Psychology of Childhood and Youth

Overview

For the following analyses, one of the three main factors of the design was reduced to one level to allow the introduction of another factor while avoiding a four-way interaction. In the case of the ratings, this factor was emotion, now reduced to separate analyses for happiness, sadness, and anger respectively. In the case of mimicry, this factor was segment, now reduced to a focused analysis of the apex segment (i.e., peak of expressivity). Therefore, these ancillary models are not directly comparable with the models from the main analyses (see manuscript) as they do not have the same factor structure. Instead, these analyses were conceived of as additional explorations into the data to uncover some potential avenues for moderator variables in future research. The predictive value of each potential moderator was assessed via likelihood-ratio tests (LRT). The model comparisons started off with the comparison between the interaction model targetage*mask*moderator M (i.e., model: DV~ targetage*mask*M). and the targetage*mask+main effect of the moderator M (i.e., model: DV~ targetage*mask+M). Depending on the moderation, several further models were estimated to carve out the moderation. The individual moderation effects were

assessed via simple slopes (package *reghelper*; Hughes, 2022). The moderator variables were:

Depression (phq_sum_cen): Clinical monitoring tool PHQ-9 (Patient Health Questionnaire, nine items; Kroenke et al., 2001). Scores each of the nine DSM-IV criteria as 0 = not at all to 3 = nearly every day. An example item is "Feeling bad about yourself - or that you are a failure or have let yourself or your family down?" As the variable did not have a meaningful zero (only a rating anchor), it was centered.
Anxiety (gad_sum_cen): Clinical screening tool GAD-7 (General Anxiety Disorder-7 Scale; Spitzer et al., 2006). Scores each of the nine DSM-IV criteria as 0 = not at all to 3 = nearly every day. Response categories: 0 = not at all, 1 = several days, 2 = more than half the days, and 3 = nearly every day. An example item is "Feeling afraid as if something awful might happen?" As the variable did not have a meaningful zero, it was centered.
Mask endorsement (meanmws_r_cen): 9-item scale, 5-point, adaptation of An's (2021) Coronavirus Social Distancing Scale to the context of mask-wearing. In each item, the term social distancing was simply replaced by the term mask wearing. The logic of the syntax and semantic of the statement were preserved in each case. An example item was "Mask wearing [Social distancing] should be a matter of personal choice." As the variable did not have a meaningful zero, it was centered.
Mask compliance (att1_cen): Single item, 5-point, item: "How often do you wear a mask in designated places (e.g., public transportation, supermarket, etc.)?" 1 never, 2 rarely, 3 sometimes, 4 often, 5 always. As the variable did not have a meaningful zero, it was centered.
Mask voluntariness (att2_cen): Single item, 5-point, item: "Would you also wear a mask if this was not mandatory?" 1 never, 2 rarely, 3 sometimes, 4 often, 5 always. As the variable did not have a meaningful zero, it was centered.
Perceived interpersonal closeness (IOS): Inclusion of Other in the Self-scale (IOS, Aron et al., 1992), adapted as a slider from 0 to 100 to achieve a continuous measure. The instruction read as follows: "When you like someone or would like to spend time with that person, you often talk about closeness. Here you can see two circles that you can move with the mouse. Imagine that one circle is you and the other circle is the person you just saw. The closer you feel to the person, the closer you move your circle over the other person's circle." Of course, the IOS scale was not used as a moderator when IOS was the DV. As the variable had a meaningful zero, it was not centered.

Results

Listed below are statements on significant effects on the dependent variables hit rate, intensity, perceived interpersonal closeness (here without IOS as moderator), emotion bias, negatively valenced distractor emotions, overall emotion profile use as well as happiness, sadness, and anger mimicry (for full results see R Markdown at https://osf.io/smqpk/?view_only=f2681fb953f54a149238efaf243bb9c0).

In the great majority of the cases, the focal targetage*mask interaction remained intact, except for all moderators in the perceived closeness model for sadness (mask main effect remained), mask compliance in the perceived intensity model for sadness (mask main effect remained), mask voluntariness in the perceived intensity model for sadness (no effect), perceived closeness in the negatively valenced distractor emotions model for happiness (mask main effect remained), anxiety, depression, mask voluntariness, and closeness in the overall emotion profile use model for sadness (mask main effect remained], anxiety, depression, mask compliance, mask voluntariness, in the overall emotion profile use model for anger (no effect). As noted above, however, the factor structures of these ancillary models were not directly comparable with the main analysis models and therefore only have exploratory purpose for future research directions. We do not infer conclusions from these moderation analyses beyond this sample. Especially in the case of main effects without any condition differences, and especially in the case of ratings, we cannot rule out simpler alternative explanations based on methodological artifact (e.g., response tendencies). Moreover, we cannot rule out power issues when there was no effect left whatsoever; thus, no further interpretation on potential buffering effects were made. These results are intended to give an insight into potential avenues for future moderator variables and into the robustness of the obtained main analysis results.

Hit Rate

For all emotions, the three mask-related attitudes: mask endorsement, mask compliance, and mask voluntariness did not moderate the hit rate results. However, there was a significant three-way interaction between perceived interpersonal closeness (IOS), target age, and mask (χ^2=71.8, p<.0001, comparison to model with only the two-way interaction plus closeness main effect). Higher closeness was associated

with better recognition of happiness, except in the case of unmasked child faces. For sadness, there was a significant two-way interaction of targetage and closeness (χ^2=71.1, p<.0001, comparison to model with only the two-way interaction plus closeness main effect). Only at the lowest level closeness, hit rates for unmasked child faces were not better than for masked ones. For anger, no moderations were found; only a main effect of IOS on hit rate (χ^2=27.5, p<.0001, comparison with mask*targetage two-way interaction model) in that more closeness was associated with lower tendency to recognize anger correctly.

Intensity

For happiness, we found a significant two-way interaction between mask and mask compliance in the three-way model (χ^2=17.1, p<.001, comparison to two-way model plus mask compliance main effect). Higher levels of mask compliance were associated with higher perceived intensity in all cases except unmasked adult faces. We also found a significant three-way interaction of target age, mask, and closeness (χ^2=147, p<.0001, comparison to model with mask*target age interaction plus closeness main effect). More closeness was associated with more perceived happiness, and this effect was stronger for masked child faces in comparison to all the remaining factor level combinations. In the case of masked adult faces, the effect was only stronger in comparison to child unmasked but not adult unmasked faces. For sadness and anger, no significant moderation was found. There was only a main effect of closeness on perceived sadness (χ^2=124, p<.0001, comparison with two-way model) and perceived anger (χ^2=28.3, p<.0001, comparison with with two-way model). The closer participants felt, the higher were the sadness but the lower were the anger ratings.

Perceived Interpersonal Closeness

A significant mask by mask compliance interaction emerged for all emotions (happy: χ^2=37.8, p<.0001, sad: χ^2=1., p=.001, anger: χ^2=21.3, p<.0001, comparison with mask main effect model). In all three cases, the plot pattern reflected the idea that closeness decreased in response to unmasked faces when mask compliance was higher, whereas the simple slopes not always confirmed this.

Emotion Bias

For happy faces, there was a mask * mask compliance interaction (χ^2=27.8, p<.0001, comparison with mask main effect model). In the masked conditions, higher mask compliance was associated with less emotion bias.

Moreover, there was a mask * closeness interaction (χ^2=171, p<.0001, comparison with mask main effect model). In the masked conditions, the negative effect of

closeness on emotion bias was slightly stronger. For sadness, there was a main effect of closeness (χ^2=27.2, p<.0001, comparison with two-way model), in that more closeness was associated with less emotion bias. For anger, there was also a main effect of closeness (χ^2=9.09, p=.003, comparison with two-way model), but in that more closeness was associated with more emotion bias.

Negative Valence Distractor Emotions

For happiness, there was a mask * anxiety interaction (χ^2=11.3, p=.004, comparison with mask main effect model). In the masked conditions, higher anxiety was associated with higher tendency to perceive negatively valenced distractor emotions.

Moreover, there was a mask * mask compliance interaction (χ^2=80.5, p<.001, comparison with mask main effect model). In the masked conditions, higher mask compliance was associated with a reduced tendency to perceive negatively valenced distractor emotions. Furthermore, there was a main effect of closeness for happiness (χ^2=213, p<.0001, comparison with two-way model) and sadness (χ^2=36.1, p<.0001, comparison with two-way model with mask by targetage), in that more closeness was associated with less perceived negatively valenced distractor emotions. Moreover, for happy (χ^2=12.9, p<.0001, comparison with two-way model with mask by targetage) and angry (χ^2=10.7, p=.001, comparison with two-way model with mask by targetage) faces, there was a main effect of mask compliance on negatively valenced distractor emotions in that the latter were reduced when mask compliance was higher.

Emotion Profile Use

For happiness, there was a mask*anxiety interaction (χ^2=13.8, p<.001, comparison with mask main effect model). In the masked conditions, higher anxiety was associated with higher tendency to use the emotion profile overall. Moreover, there was a targetage*mask*closeness three-way interaction (χ^2=28.3, p<.001, comparison with two-way interaction model plus closeness main effect). The effect of closeness on overall emotion profile use was stronger for child masked faces than for child and adult unmasked faces.

There was a main effect of anxiety in that higher anxiety scores were associated with less overall emotion profile use in the case of sadness (χ^2=7.26, p=.007, comparison with two-way model) and anger (χ^2=7.24, p=.007, comparison with two-way model with mask by targetage), most likely a response tendency. There was also a main effect of closeness in that higher closeness was associated with more emotion profile use for sad faces (χ^2=9.86, p=.002, comparison with two-way model with mask by targetage).

Facial Mimicry

Happy Expressions

No moderation effects were found (focused analysis for apex segment) but there was a main effect of IOS (χ^2=34.9, p<.0001, comparison with two-way model with mask by targetage), such that higher closeness was associated with less mimicry, which according to the plot pattern appeared mostly in the masked condition, whereas the difference was not significant.

Sad Expressions

There was a main effect of mask compliance (χ^2=8.85, p<.0001, comparison with two-way model with mask by targetage). Higher mask compliance was associated with more facial negativity in response to sad faces.

Angry Expressions

There were no moderation effects.

For more details, please see R Markdown.
